# Tillage effects on soil properties and crop yield after land reclamation

**DOI:** 10.1038/s41598-021-84191-z

**Published:** 2021-02-25

**Authors:** Zhe Liu, Shiliu Cao, Zenghui Sun, Huanyuan Wang, Shaodong Qu, Na Lei, Jing He, Qiguang Dong

**Affiliations:** 1 Shaanxi Provincial Land Engineering Construction Group Co., Ltd., Xi’an, 710075 China; 2 Key Laboratory of Degraded and Unused Land Consolidation Engineering, The Ministry of Natural Resources, Xi’an, 710075 China; 3 Institute of Land Engineering and Technology, Shaanxi Provincial Land Engineering Construction Group Co., Ltd., Xi’an, 710075 China; 4 Shaanxi Provincial Land Consolidation Engineering Technology Research Center, Xi’an, 710075 China

**Keywords:** Environmental sciences, Ecology, Agroecology

## Abstract

Tillage treatments have an important effect on soil microstructure characteristics, water thermal properties and nutrients, but little is known in the newly reclaimed cultivated land. For the reason, a long-term field study was to evaluate the tillage effects on soil physicochemical properties and crop yield in newly reclaimed cultivated land via the macroscopic and microscopic analysis. Three tillage treatments were tested: continuous conventional moldboard plow tillage (CT), sub-soiling/moldboard-tillage/sub-soiling tillage (ST) and no-tillage/sub-soiling/no-tillage (NT). Under CT, the microstructure was dominated by weakly separated plates structure and showed highest bulk density (BD) (1.49 g cm^−3^) and lowest soil organic matter (SOM) (3.68 g kg^−1^). In addition, CT reduced the capacity of soil moisture retention and temperature maintenance, resulting in aggregate structure deterioration and fragility. Unlike CT, the soil was characterized by moderately separated granular structure and highly separated aggregate structure under conservation tillage practice of ST and NT. NT was associated with the highest soil moisture content (20.42%), highest quantity of macroaggregates (> 0.25 mm) by wet-sieving (34.07%), and highest SOM (6.48 g kg^−1^) in the surface layer. Besides, NT was better able to regulate soil temperature and improved the values of geometric mean diameter. Under NT and ST, a stable soil structure with compound aggregates and pores was formed, and the maize yield was increased by 12.9% and 14.9% compared with CT, up to 8512.6 kg ha^−1^ and 8740.9 kg ha^−1^, respectively. These results demonstrated the positive effects of NT and ST on soil quality and crop yield in newly reclaimed cultivated land.

## Introduction

With the rapid development of China’s industrialization and urbanization, the amount of cultivated land decreases sharply, and the conflict between people and cultivated land is intensifying, which will threaten food security and healthy development of agriculture. In order to curb the reduction in the amount of cultivated land, China has launched a large number of newly reclaimed cultivated land projects to supplement cultivated land resources in a timely manner^1–3^. The newly reclaimed cultivated land is an important cultivated land resource, which was formed by covering raw soil on barren and abandoned gravel land after land reclamation. Among the land resources that can be developed and utilized in China, barren and abandoned gravel land accounts for 32.31%, which has the characteristics of covering a larger area and having great potential for development^4^. After transformed into newly reclaimed cultivated land via relevant engineering and agricultural management measures on barren gravel land, it is not only of great importance to contribute to regional food security and healthy development of agriculture, but also beneficial to ecological safety and economic development^5^. Although the amount of cultivated land has been supplemented, the newly reclaimed cultivated land is different from the traditional cultivated land soil. The problems of newly reclaimed cultivated land, such as low soil maturity, poor soil structure and low productivity, severely limit the availability of newly cultivated land^5–7^. Therefore, increasing productivity by improving the quality of newly reclaimed cultivated land is extremely important^7,8^. Many different fertilization measures had been used to improve soil quality of newly reclaimed cultivated land, and research results showed that organic fertilizer application with chemical fertilizer was the best choice for soil improvement^6^. Fly ash, phosphogypsum and other amendments have been adopted to restore the soil nutrient status of newly reclaimed cultivated land, which concluded that mineral addition had a positive impact on soil water thermal properties, soil nutrients and crop yield^9,10^. In addition, previous studies have also shown that some field engineering measures such as land leveling, cultivated land restructuring and irrigation and water conservancy construction play a certain role in improving soil quality^11^. However, these current measures mainly focus on the optimization of fertilization methods, the selection of soil mineral modifiers and land consolidation to improve newly reclaimed cultivated land soil quality. Tillage treatment is a traditional soil improvement method, which can significantly regulate the dynamic balance of water, fertilizer, gas and heat in the soil^12^. It also affects the structural stability of the soil and the absorption of nutrients by crops, and finally affects crop yield^13^. In particular, conservation tillage practices such as no-tillage, subsoiling tillage and straw mulching have achieved remarkable results in increasing soil nutrient content, improving soil structure, and reducing soil erosion, which is of great significance to ensure sustainable development of soil and agricultural ecological environment^14–16^. However, there are few studies on the effects of different tillage treatments on newly reclaimed cultivated land, which seriously affects the healthy development of newly reclaimed cultivated land.

In recent years, inappropriate agricultural practices, such as intensive and conventional tillage systems that leave soil surfaces bare are main driving forces for agricultural soil degradation^14^. Conservation tillage practices such as no-tillage and straw mulching have been paid more and more attention. It has been observed that subsoiling tillage treatment can effectively break the plow pan of cultivated land soil, improve soil structure and increase soil porosity and water retention capacity^17,18^. The effect of no-till treatment on soil bulk density is not as obvious as that of ST. Advantages of stubble mulching no-till are related to SOM, the activity of microorganisms, the number of aggregates and the stability of soil structure, which can create stable cultivation layer that will be conducive to the growth of crops^16,19–21^. However, due to intensive mouldboard plow tillage, the soil structure under the conventional tillage practices is deteriorated, which has caused the decline of soil quality and lower and unstable crop yields^22,23^. Previous research on tillage treatments has focused on the soil improvement of traditional cultivated land^15,18^. Newly reclaimed cultivated land is constructed with raw soil as the main material, which is different from the traditional cultivated land soil. After land reclamation, the soil structure of the newly reclaimed cultivated land is unstable and relatively fragile^5^. Inappropriate single tillage treatment can cause soil particles to migrate and settle, accelerate the nutrients mineralization and depletion, reduce the number and structural stability of aggregates and disrupt surface vented pores, resulting in the compaction of lower layer soil and the formation of plow pan^24^. Due to soil compaction and settling, the physical and chemical properties and crop growth will be greatly affected. Therefore, the sustainable tillage practice of soil in the newly reclaimed cultivated land is very important, yet few studies have compared the characteristics of newly reclaimed soil microstructure and physicochemical properties under different tillage treatments from a microscopic perspective, it is necessary to carry out further research about the improvement by different tillage treatments on the newly reclaimed cultivated land. At present, new equipment based on environmental scanning electron microscope (SEM) plays an important role in revealing soil microstructure characteristics and dynamic evolution, and the further study of soil microstructure by using SEM helps to understand soil properties and internal mechanisms^25–27^.

We hypothesized that conservation tillage practices with NT and straw mulching could influence soil physicochemical properties in newly reclaimed cultivated land, significantly increase soil water content and crop yields by improving soil structure and increasing soil organic matter content, thereby enhancing soil productivity. However, the information on the influence of different tillage practices in newly reclaimed cultivated land is scarce. In order to make up for this lack of information about the effect of different tillage treatments on the newly reclaimed cultivated land, the objective of this study was to evaluate the: (1) effects of three different tillage treatments on soil organic matter, aggregate distribution and stability, soil temperature, soil bulk density and soil water content; (2) soil microstructure characteristics under three different tillage treatments by using SEM; and (3) effects of different tillage treatments on crop yield in newly reclaimed cultivated land. The research results will provide a theoretical basis for selecting suitable tillage measures for soil quality improvement in newly reclaimed cultivated land.

## Results and discussion

### Effects of different tillage treatments on soil properties

#### Soil organic matter (SOM) and total nitrogen (TN)

**Figure 1:**
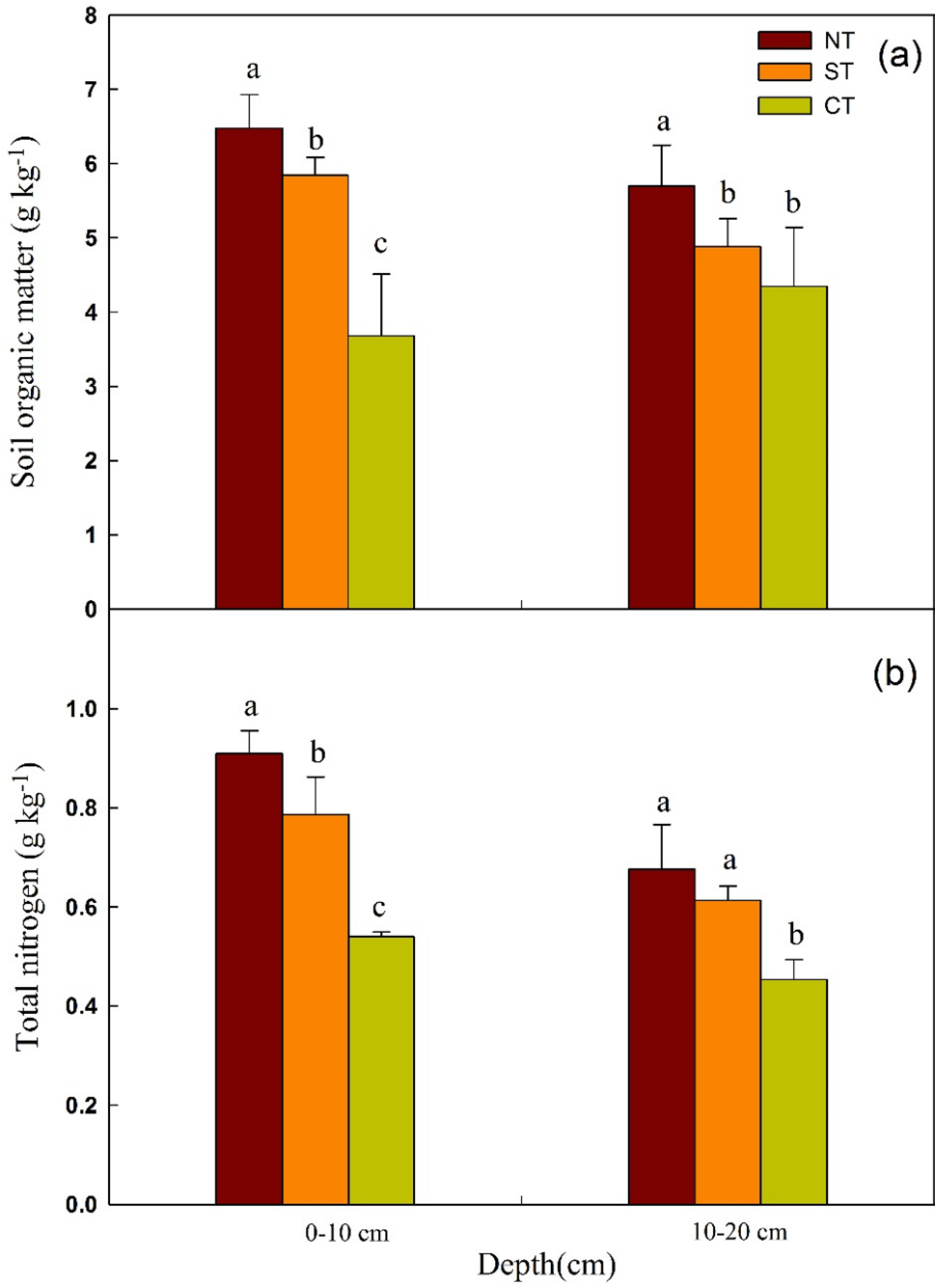
SOC and TN concentration in different tillage treatments. *CT* continuous conventional moldboard-tillage, *NT* no-tillage/sub-soiling/no-tillage, *ST* sub-soiling/moldboard-tillage/sub-soiling tillage, *SOM* soil organic matter, *TN* total nitrogen. Different lowercase letters represent significant differences between different tillage treatments in the same soil layer.

SOM and TN have positive effects on soil physicochemical properties and crop productivity. SOM can not only increase soil nutrients, soil aggregation, aggregate stability and soil C conservation, but also improve soil water storage capacity and promote crop growth^28–30^. TN plays an important part in improving soil fertility, promoting plant residues decomposition, increasing crop yield, and promoting the coordination of water and fertilizer^31,32^. The effect of tillage treatments on SOM and TN is shown in Fig. 4. The content of SOM under NT and ST was 36.4% and 22.9% higher than that under CT (*P* < 0.05) at the 0–10 cm layer, 31.1% and 12.3% higher at the 10–20 cm (Fig. 1a). Under NT treatment, the content of SOM was significantly higher than that under ST and CT treatment, while no significant difference was observed between ST and CT treatment at 10–20 cm. It could be clearly seen from Fig. 1, the concentration of SOM under NT and ST gradually decreased with the increasing soil depth, while increased with depth under CT. It was plausible that CT changed the spatial distribution of the soil layer and accelerated SOM mineralization at surface layer. Result for the TN content was similar to SOM (Fig. 1b). At the two soil layers, the TN concentration under NT and ST was markedly increased, compared with CT, and the increase at 0–10 cm was 68.5% and 45.7%, respectively.

In summary, CT with frequent tillage operations disturbed soil greatly and destabilized soil structure, which may accelerate the nutrients mineralization and decomposition^33^. In addition, CT exposed soil surfaces by removing crop residues, decreased the C input through reducing the recycling of crop residues, which led to the loss and depletion of nutrients at the surface layer. In contrast, the soil disturbance was slight under NT and ST with crop straw residues covered, high activity of microorganisms and enzymes may accelerate the decomposition of crop residues, and increased C input through recycling of crop residues. Moreover, NT and ST reduced soil disturbance and mechanical compaction, decelerated the mineralization and degradation of soil nutrients, which was helpful to increase the content of SOM and TN and improve soil quality^34^. Therefore, the result was consistent with previous studies, which reported no-tillage treatment had a higher SOM and TN concentration compared with conventional tillage at the surface layer^35–37^.

#### Size distribution of aggregates

**Figure 2:**
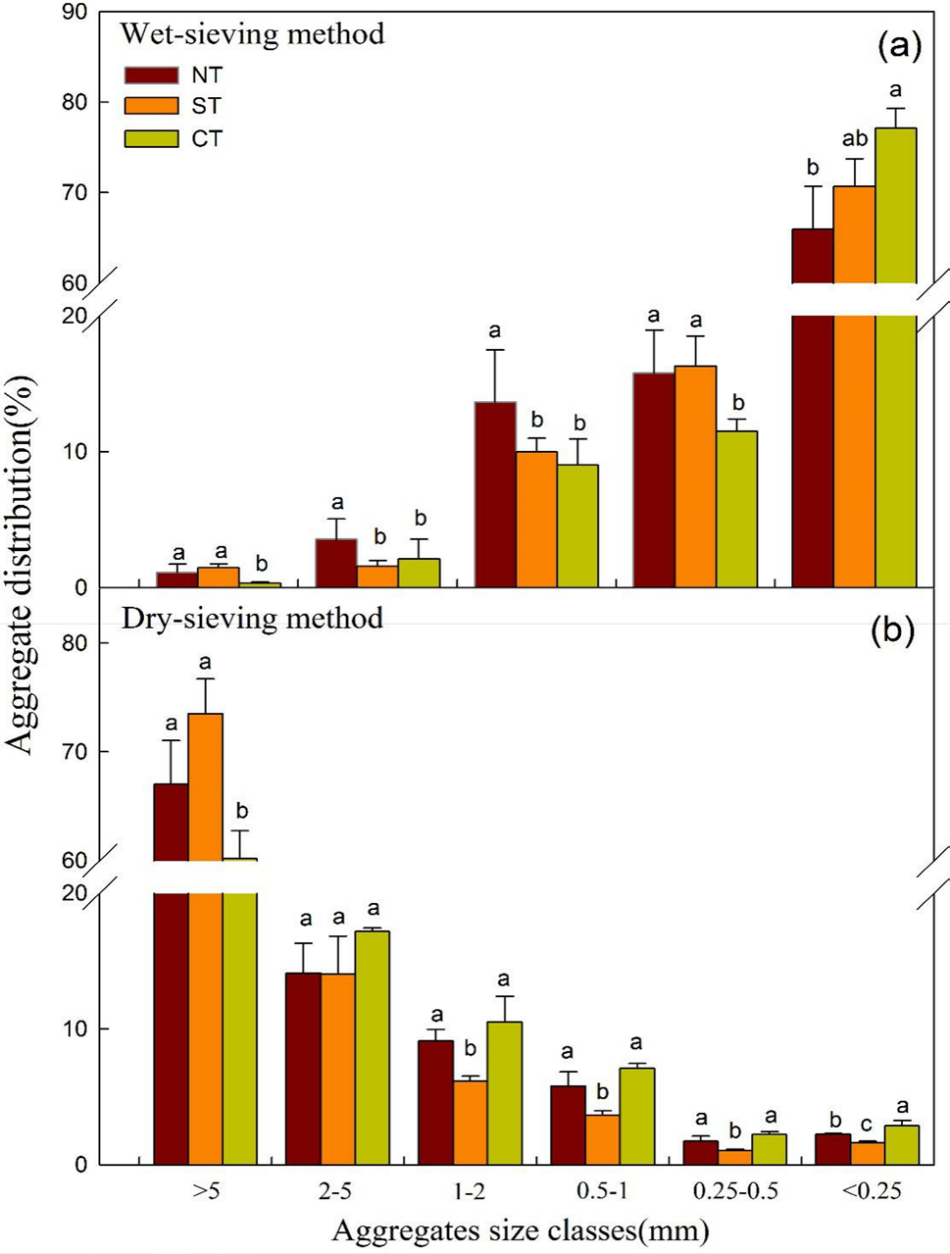
Aggregate size distribution under different tillage treatments. *CT* continuous conventional moldboard-tillage, *NT* no-tillage/sub-soiling/no-tillage, *ST* sub-soiling/moldboard-tillage/sub-soiling tillage. Different lowercase letters represent significant differences between different tillage treatments in the same particle-size aggregates.

**Figure 3:**
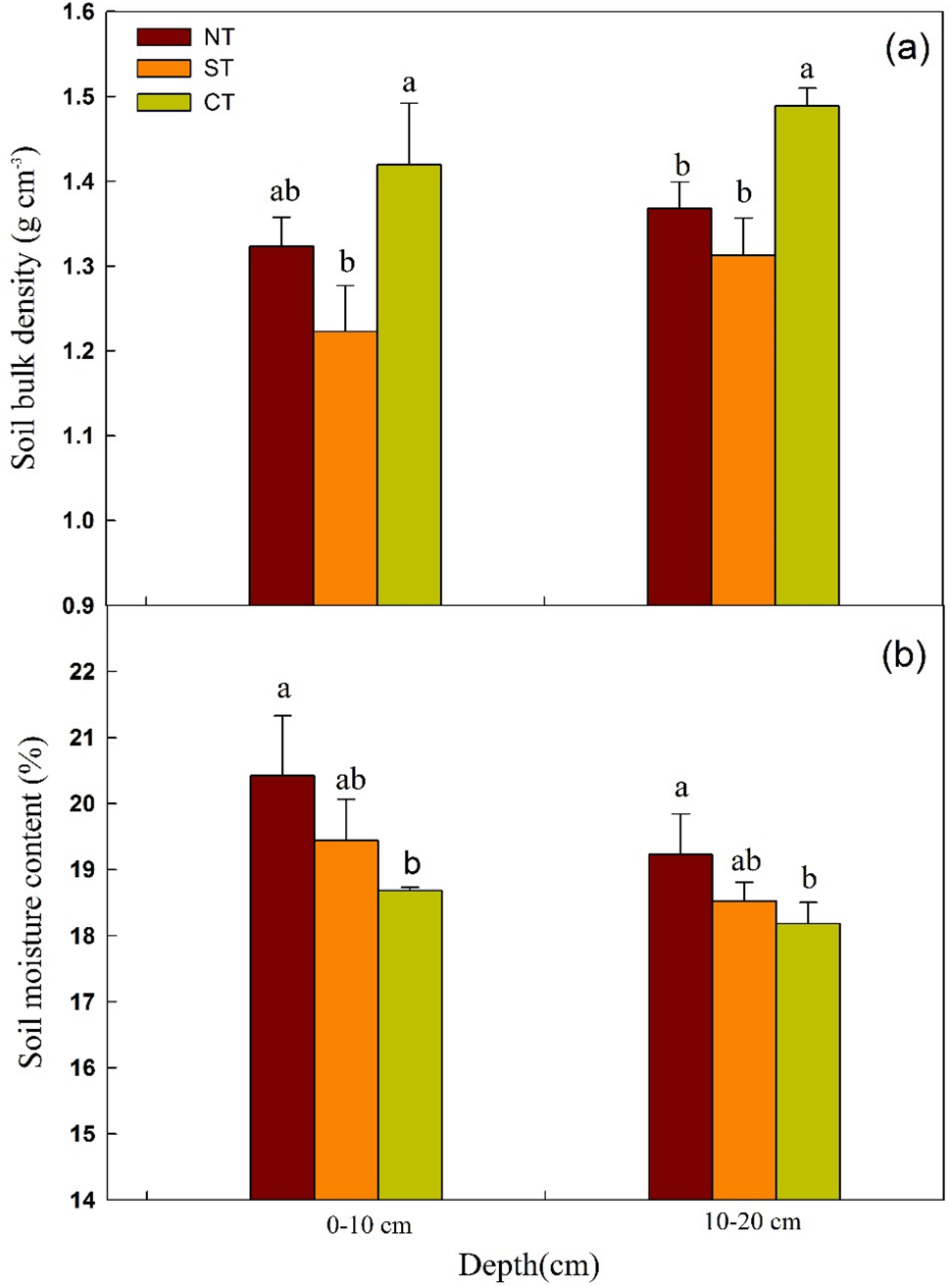
Comparison of tillage treatments on BD and SMC at different soil layers. *CT* continuous conventional moldboard-tillage, *NT* no-tillage/sub-soiling/no-tillage, *ST* sub-soiling/moldboard-tillage/sub-soiling tillage, *BD* soil bulk density, *SMC* soil moisture content. Different lowercase letters represent significant differences between different tillage treatments in the same soil layer.

Different tillage treatments have different effects on the distribution of different particle-size aggregates (Fig. 2). Compared with CT, the distribution data of aggregates determined by the wet-sieve method showed that NT significantly increased the proportion of > 0.25 mm aggregates, and decreased the proportion of < 0.25 mm aggregates (Fig. 3a, P < 0.05). For water-stable macroaggregates (> 0.25 mm), NT and ST increased by 48.8% and 28.0% compared with CT, respectively. NT showed a significant effect on the distribution of different particle-size aggregates, which was verified by studies of Du et al. and Bottinelli et al.^38,39^. The size distribution of aggregates determined by dry-sieve method showed that NT and ST significantly increased the proportion of > 5 mm particle-size aggregates and decreased the proportion of < 0.25 mm aggregates (Fig. 3b). No significant difference was observed on the proportion of other particle-size aggregates. Compared with CT, the proportion of macroaggregates under NT and ST increased by 6.3% and 12.8%, respectively.

In summary, due to soil disturbance in different degrees and different straw treatment methods, different tillage treatments have different influence on the formation of different particle-size aggregates. The results of dry-sieve method and wet-sieve method suggested that both NT and ST increased the proportion of macroaggregates and reduced the aggregate breakdown. It may indicate that conservation tillage practices and application of crop residues decrease soil disturbances and increase SOC content, which is conducive to the formation and stability of soil aggregates. These findings were supported by the results of Bhattacharyya et al., who found that NT was beneficial to the formation of > 0.25 mm macroaggregate fractions compared with CT at the surface layer of soil. SOM is the main cementing agent for soil aggregates, which is known to play a key role in soil aggregation^40,41^. However, due to intensive tillage and low SOC content, CT accelerated the reduction of > 0.25 mm macroaggregate fractions and increased the proportion of microaggregates (< 0.25 mm)^16,19^.

#### Stability index of soil aggregates

The results of dry-sieve method and wet-sieve method showed that geometric mean diameter (GMD), mean weight diameter (MWD), > 0.25 mm macroaggregates (*R*_0.25_) values under NT and ST were higher than that under CT, while the fractal dimension (*D*) value was lower than CT (Table 1). Compared with CT, the results of the wet sieve method showed that the MWD, GMD, and *R*_0.25_ values were increased by 17.9%, 11.8%, and 48.8% under NT treatment and 7.2%, 4.5% and 28.0% under ST treatment, respectively. NT had the most obvious effect on the stability index of aggregates. Soil structural stability depends on the proportion of stable aggregates, and a decrease in proportion and stability of macroaggregates may affect soil structural stability^42–44^. GMD, MWD, *R*_0.25_ and *D* are important indicators to evaluate the stability and distribution of soil aggregates, higher GMD, MWD and *R*_0.25_ values, and lower *D* value indicate higher soil particle aggregation and better soil structural stability^45,46^. Therefore, compared with CT, both NT and ST can enhance the soil agglomeration and structural stability. Due to the minimum disturbance of soil aggregates, NT showed the greatest effect on improving soil structure^40,46^.

**Table 1:** Comparison of different tillage rotation treatments on aggregate stability index.

Method	Treatments	MWD (mm)	GMD (mm)	*D*	*R*_0.25_ (%)
Dry-sieving	NT	4.04 ± 0.11b	3.41 ± 0.14b	2.19 ± 0.09a	97.8 ± 0.06b
	ST	4.29 ± 0.06a	3.82 ± 0.07a	2.02 ± 0.09b	98.4 ± 0.12a
	CT	3.84 ± 0.09c	3.14 ± 0.11c	2.33 ± 0.05a	97.2 ± 0.41c
Wet-sieving	NT	0.40 ± 0.02a	0.34 ± 0.01a	2.93 ± 0.01b	34.1 ± 4.75a
	ST	0.37 ± 0.01ab	0.32 ± 0.01ab	2.96 ± 0.01ab	29.3 ± 3.04b
	CT	0.34 ± 0.02b	0.30 ± 0.01b	2.97 ± 0.02a	22.9 ± 2.19c

*GMD* geometric mean diameter, *MWD* mean weight diameter, *D* fractal dimension, *R*_*0.25*_ > 0.25 mm aggregates, *CT* continuous conventional moldboard-tillage, *NT* no-tillage/sub-soiling/no-tillage, *ST* sub-soiling/moldboard-tillage/sub-soiling tillage.

Different lowercase letters represent significant differences between different tillage treatments in the same aggregate determination method.

#### Soil bulk density and soil water content

The soil bulk density (BD) under the ST and NT was lower than that under CT to a greater extent at the 0–20 cm soil depth layers, however, the difference was not significant under the NT and CT at the 0–10 cm (Fig. 3a). BD under ST and NT was reduced by 13.8% and 6.8% at the 0–10 cm soil layer, and 11.8% and 8.1% at the 10–20 cm, respectively, compared with CT. The minimum soil bulk density was observed under ST treatment with a value of 1.22 g cm^−3^, and due to intensive tillage operations and straw removal, the maximum BD was observed under CT with a value of 1.49 g cm^−3^. At the 0–10 cm, significant differences were found in BD between ST and CT (*P* < 0.05), while no significant difference between NT and CT. Significant differences were observed between CT and ST treatment (*P* < 0.05), while no significant difference between ST and NT at the 10–20 cm. The value of BD was higher at 10–20 cm than that at the 0–10 cm soil layer, especially in the lower soil under CT. Specifically, ST remarkably reduced the surface BD. NT was also found to result in lower BD than CT slightly, which was consistent with the result of Blanco et al., who found a decrease in BD in the surface layer under NT treatment^47^. The slight decrease in BD under NT treatment compared with CT, which may be partially attributed to the increase in SOM content resulting from application of crop residues and improvement effect of NT coupled with subsoiling. BD is directly affected by SOM, as the particle density of SOM is considerably lower than that of mineral soil^48^. Rintaro et al. reported a lower BD in soils with higher levels of SOM content under NT treatment, which may partly explain our result^49^. As discussed above, compared with CT, NT and ST played a certain role in reducing the BD, which is likely caused by over disturbance to the soil without crop straw under CT. However, CT can lead to the migration of small soil particles, blockage in pores, and the reduction of crop residues and organic matter content at the 0–20 cm tillage layer, which causes soil compaction and increases the BD in newly reclaimed cultivated land^14,19^.

Soil water has important effects on the formation and development of soil, the movement of materials and energy in the soil and the growth of crops. Among the three tillage treatments, NT resulted in the largest soil moisture content (SMC), following by ST and CT at the 0–10 cm and 10–20 cm soil layers (Fig. 3b). At the 0–10 cm soil layer, the SMC under NT and ST was increased by 9.9% and 4.1% compared with CT, and significant difference was observed between NT and CT (*P* < 0.05). At the 10–20 cm soil layer, The SMC of ST and NT was 5.8% and 2.0% more than that under CT, and the difference between NT and CT treatment was significant (*P* < 0.05). In contrast, no significant difference was observed between ST and CT. The results indicated that both NT and ST could improve soil water storage when compared with the CT while the poor water storage under CT was prone to soil moisture loss. The plausible explanation is that CT increases the frequency of tillage with straw removed, resulting in soil compaction and less water transmission and retention. It is similar to the research results of Zhou et al., who indicated that intensive conventional tillage would hinder the water infiltration^24^. However, conservation tillage practices of NT and ST reduced tillage intensity and preserved straw cover that increased soil water infiltration and SMC, and displayed a high water-holding capacity^50^.

#### Soil temperature

**Figure 4:**
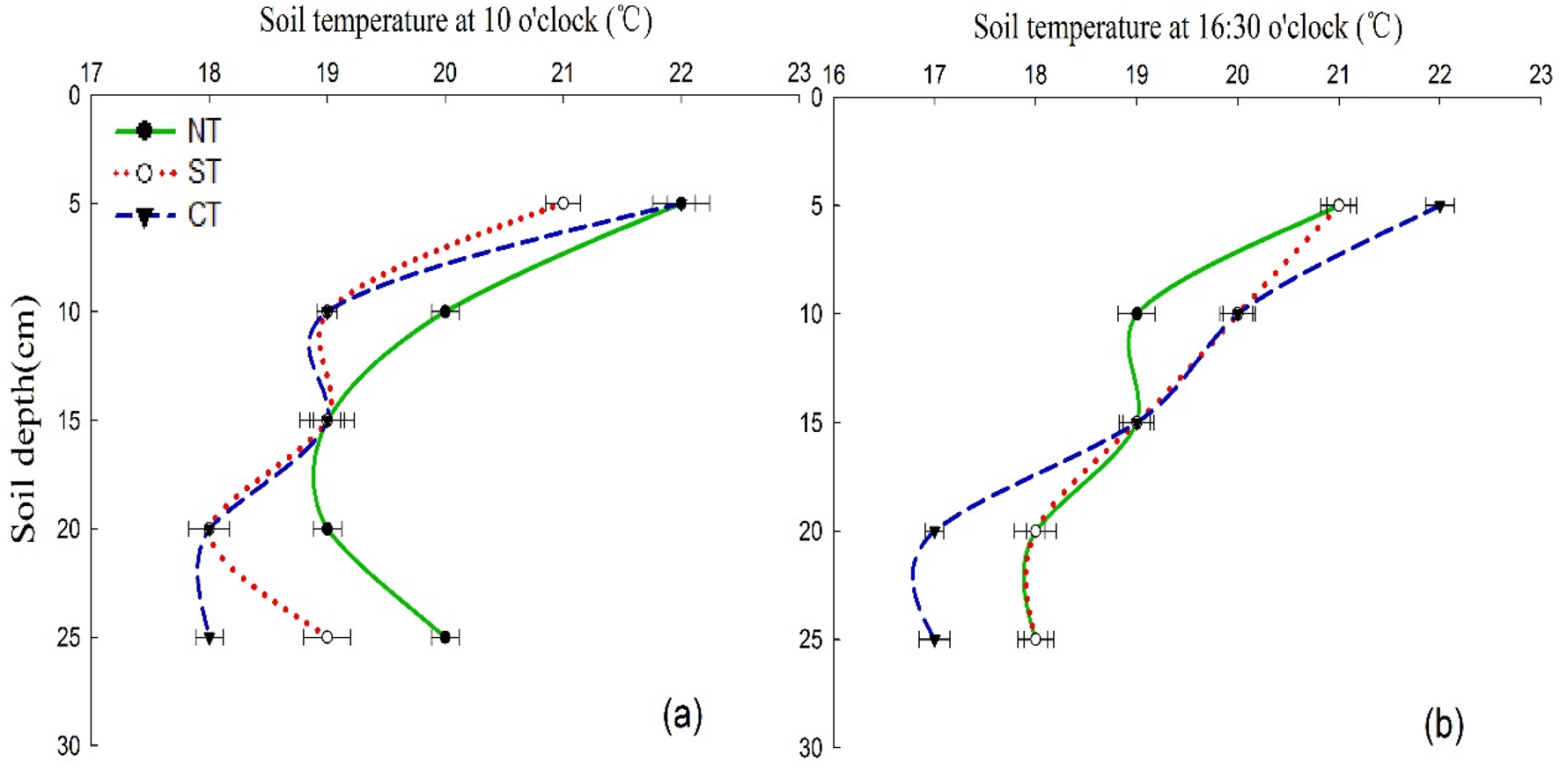
Comparison of tillage treatments on soil temperature at different soil layers. *CT* continuous conventional moldboard-tillage, *NT* no-tillage/sub-soiling/no-tillage, *ST* sub-soiling/moldboard-tillage/sub-soiling tillage.

Soil temperature is a comprehensive indicator of soil thermal conditions, which affects the availability of soil nutrients and crop growth and development. With the increase of soil depth, the soil temperature showed a trend of decreasing first and then gradually stabilizing under all tillage treatments in general, except for the soil temperature at the 25-cm layer under NT and ST at 10 o’clock (Fig. 4). The air temperature was 16.2 °C and 22.8 °C at 10 o’clock and 16:30 o’clock, respectively. As shown in Fig. 4, the variation extent of soil temperature under NT and ST was smaller compared with CT at both two time points, thus indicating a poor ability to regulate soil temperature under CT. The high temperature in the 20–25 cm soil layer under NT and ST treatments, which may be partially attributed to lagging effect of soil temperature conduction and straw mulching^51,52^. Meanwhile, the soil temperature under NT was generally higher than that under CT and ST at 10 o’clock (Fig. 4a), and the results was partially consistent with the results of Awe et al.^52^, who found that soil temperature was higher under no tillage compared with tilled soil. The magnitude of soil temperature variation was highest under CT at 16:30 o’clock (Fig. 4b). The possible reason was that CT increased the disturbance to the soil and removed the crop straw, resulting in a large change in soil temperature, which indicated a weak ability to regulate and maintain temperature under CT. However, NT and ST minimized soil disturbance and temperature variation, which will be better able to regulate and maintain soil temperature. Similarly, Blanco-Canqui et al. showed that NT could mitigate near surface soil temperature^53^.

### Analysis of soil microstructure characteristics based on SEM

**Figure 5:**
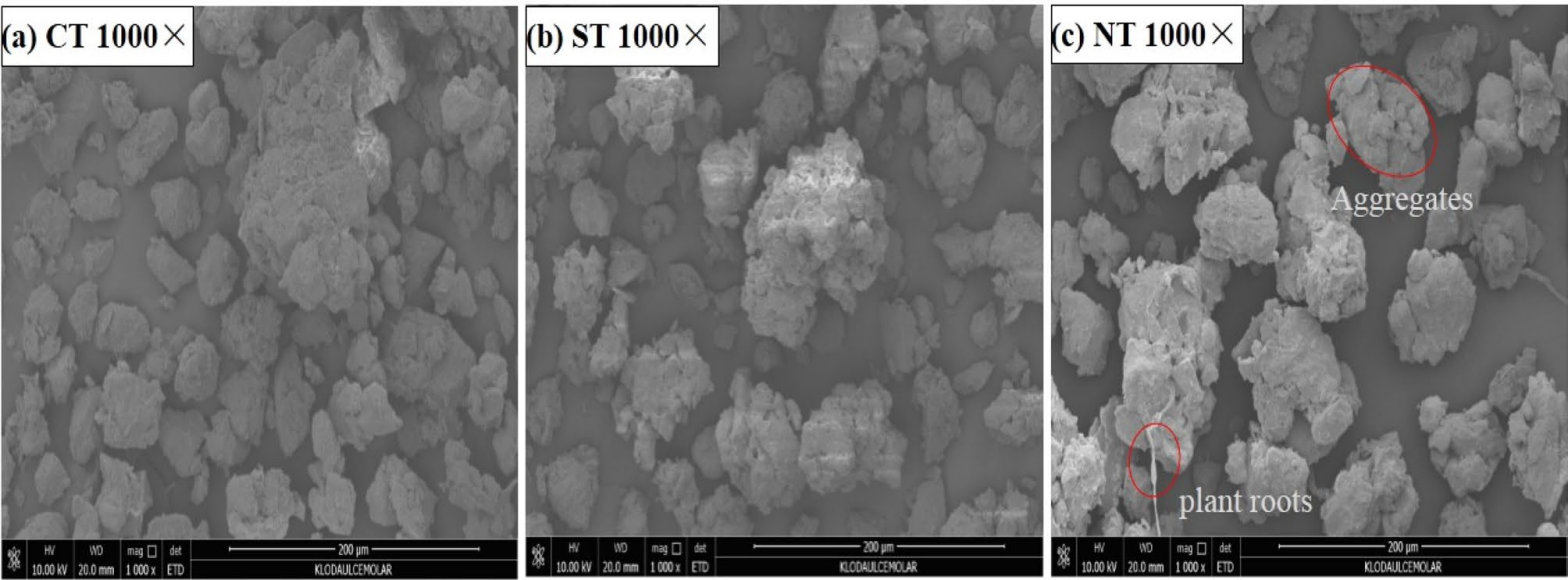
SEM images of soil under different tillage treatments. *CT* continuous conventional moldboard-tillage, *NT* no-tillage/sub-soiling/no-tillage, *ST* sub-soiling/moldboard-tillage/sub-soiling tillage, *SEM* environmental scanning electron microscope. Images were observed at the voltage of 10 kV with 1000 magnification times.

SEM is a new method to describe soil micromorphological characteristics, which is of great significance to the study of soil structure and soil fertility^26,54^. The SEM images showed that the soil microstructure under different tillage treatments changed significantly (Fig. 5). Under CT, the soil structure appears as moderately separated blocks and weakly separated plates. The main types of pores were fissures and simple accumulation pores, while composite pores were less. Furthermore, large macroaggregates, and more microaggregates and single particles were found in the soil (Fig. 5a). Under ST, the soil structure included moderately separated clumpy structures and a few highly separated granular structures. A small amount of composite packing voids and macroaggregates were found in the soil, and few fresh plant residues were observed (Fig. 5b). Under NT, the surface soil was mainly composed of moderately separated granular structures and highly developed aggregate structures. There was an abundance of well-developed aggregates and visible composite pores. Some fresh plant residues and organism traces were found in the tillage layer (Fig. 5c). Micromorphological observations and SEM images analysis suggested that NT significantly improved soil structure. This was because no-tillage with crop straw cover improved the activity of microorganisms and plants in the soil, and promoted the root growth and formation of well-developed aggregates^16,21^. Similar to NT, ST also could improve soil structure. Under ST, the number of soil aggregates increased to a certain extent based on SEM images observation and analysis, which were promoted by slight disturbance to soil and an increase in SOM. Our results were supported by the results of Zhang et al., who found that ST can stimulate the formation and preservation of aggregates^55^. However, under CT, due to excessive tillage, no crop straw was covered on the surface soil, which greatly reduced the biomass and SOM produced by cover crop straw residues, disturbed soil aggregates, accelerated the SOM mineralization and depletion^56,57^. Therefore, CT was not conducive to the formation of aggregates and the improvement of soil structure^22,23^. This was also consistent with the results of aggregate stability index, in which NT showed a good structure of highly agglomeration (Table 1).

### Effects of different tillage treatments on crop yield

Maize yields of different tillage treatments are compared in Table 2. As it can be seen, the order of maize yield was ST > NT > CT. Maize yield under ST and NT was significantly higher than CT treatment (*P* < 0.05), and little difference was observed between NT and ST. The order of average kernels per spike and kernel weight was as the same as maize yield, highest in ST, following by NT and CT. Compared with CT, the 1000-kernel weight increased by 8.5% and 4.6% under ST and NT treatments, and the maize yield increased by 14.9% and 11.9%, respectively. Compared with CT, the conservation tillage treatments of NT and ST are promising practices because they could provide good moisture, nutrient and structural conditions for high and stable crop yields, which were confirmed by the results of the previous effects on BD, SMC, aggregate structure and SOM.

**Table 2:** Maize yield under different tillage treatments.

Treatments	Row number	Kernels/row	Kernels/spike	1000-kernel weight (mg)	Effective panicles numbers	Theoretical yield (Mg ha^−1^)
NT	14.0 ± 0.63a	31.8 ± 1.46a	438 ± 27.8a	315 ± 1.13b	239 ± 1a	8512.6a
ST	12.8 ± 0.49ab	35.8 ± 2.58a	452 ± 18.1a	326 ± 0.58a	230 ± 3a	8740.9a
CT	12.4 ± 0.40b	34.6 ± 1.50a	414 ± 16.5a	301 ± 0.86c	237 ± 2a	7605.7b

*CT* continuous conventional moldboard-tillage, *NT* no-tillage/sub-soiling/no-tillage, *ST* sub-soiling/moldboard-tillage/sub-soiling tillage.

Different lowercase letters represent significant differences between different tillage treatments in the same indicator.

In summary, NT and ST minimized soil disturbance and covered crop straws compared with the CT, which was helpful to improve soil structural stability, and enhance soil water infiltration and water retention capacity. In addition, NT and ST was also better able to maintain the soil temperature, and increase SOM and TN content, which resulted in maize grain development and maize yield increasement. In contrast, CT had a relatively great disturbance to the soil and also removed crop straws, these factors accelerated the loss of soil moisture and nutrients, deteriorated the structural stability and reduced the capacity of holding soil water and fertilizer, which showed negative effects on crop aeration status and root development, resulting in unfavorable conditions for maize growth and a consequent yield reduction^58,59^.

## Conclusions

Compared with CT, the conservation tillage practice of NT and ST increased the soil water storage while reducing soil temperature variability and the soil bulk density in the newly reclaimed cultivated land. Based on SEM, the soil microstructure under NT and ST changed from moderately separated block structures to highly developed aggregate structures, and a large number of well-developed soil aggregates were formed. NT was also associated with optimal GMD, MWD, *R*_0.25_ and *D* values, which confirmed the formation of well-developed aggregates and the improvement of structural stability. In addition, NT and ST promoted the increase of SOM and total nitrogen content, improved soil structure and increased crop yields in newly reclaimed cultivated land. The study indicates that NT and ST have positive effects on soil water thermal properties, structural stability, and nutrients in newly reclaimed cultivated land, which is beneficial to high soil quality and crop yield.

## Materials and methods

### Description of the experimental site

**Figure 6:**
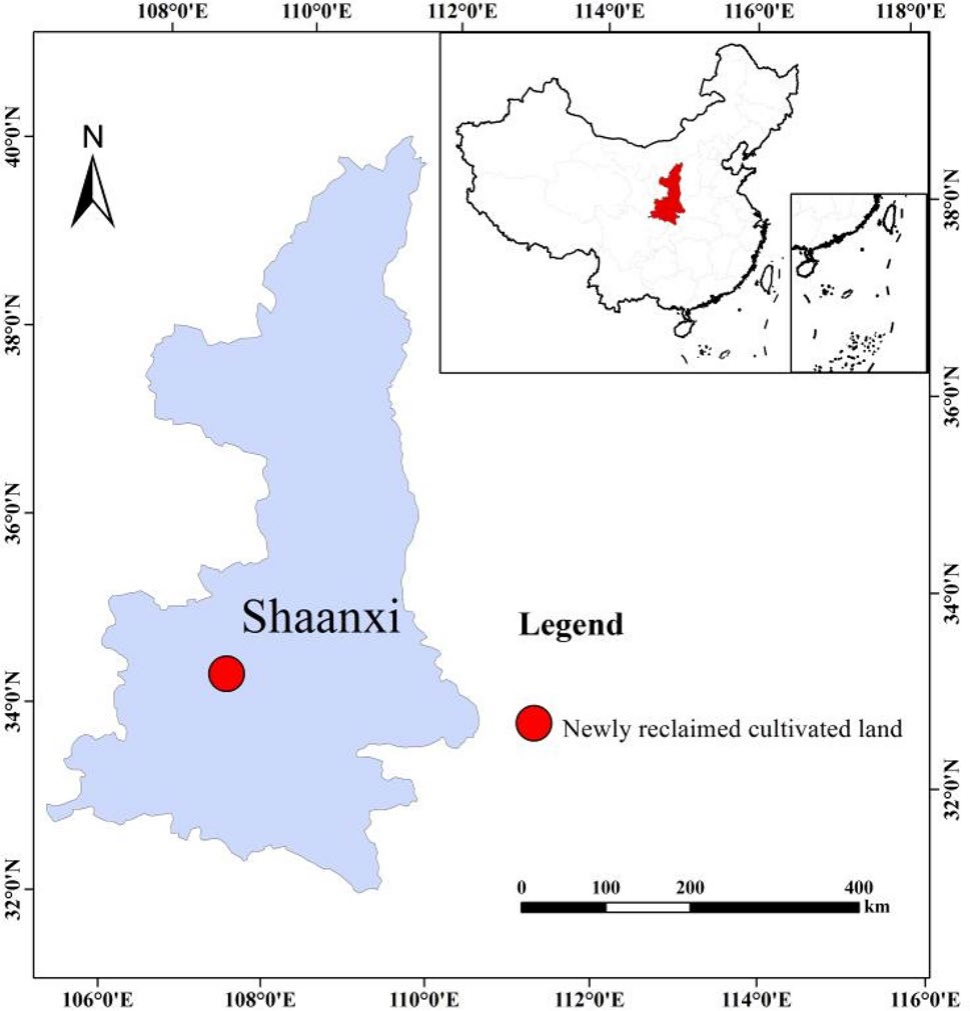
Location maps of the sampling site in the Shaanxi Province The map was produced using ESRI ArcGIS software (version 10.3; http://www.esri.com/sofware/arcgis/arcgis-for-desktop).

This study was conducted at the long-term research site of Qinling Field Monitoring Center Station (33° 59′–34° 19′ N, 107° 39′–108° 00′ E) located in Shangwang Village, Tangyu Town, Meixian County, west of Shaanxi Province, China (Fig. 6). The climate in this area is characterized by a sub-humid warm temperate continental monsoon climate. The mean annual temperature is 12.9 °C, with annual average sunshine hours of 2015.2 h. Its long-term average annual precipitation is 609.5 mm and frost-free period is 218 days. At the 0–20 cm soil layer, the soil texture type of newly reclaimed cultivated land is silt loam according to the USDA classification system, in which sand (0.05–2 mm), silt (0.002–0.05), and clay (< 0.002 mm) accounted for 4.75%, 82.99%, and 12.26%, respectively. The basic soil properties are as follows: pH value, 8.25; organic matter content, 4.07 g kg^−1^; total nitrogen content, 0.50 g kg^−1^; available phosphorus content, 20.44 mg kg^−1^; and available potassium content, 138.11 mg kg^−1^.

### Experimental design

Experiments in newly reclaimed cultivated land plot were designed to study the effects of different tillage practices on soil physicochemical properties and crop yields. The newly reclaimed cultivated land is an important available cultivated land resource, which was formed by covering raw soil from loess platform in Shangwang Village on barren gravel land after land reclamation, and barren gravel land is a widely distributed type of unused land^5^. Different tillage treatment experiments were conducted in June 2016 with cropping system of maize (*Zea mays* L.) and wheat (*Triticum aestivum* L.) rotation. The tillage experiment was arranged in a randomized complete block design with 3 replicates, and detailed tillage treatments and specific measures were shown in Table 3. There were a total of 9 experimental plots with an area of about 297 square meters, and each tillage experimental plot was 6 m long and 5.5 m wide. The tested summer maize variety was Hudan No. 4, the planting time was around June 10, the plant spacing was 30 cm, the row spacing was 50 cm, and the planting density was 6.67 × 10^4^ plants ha^−1^. The wheat variety was Xiaoyan 22, the planting time was early October, the planting density was 150 kg·ha^−1^, and the row spacing was 20 cm. Other field management procedures, such as fertilization and irrigation methods, were identical under the three tillage practices. The type of fertilizers was urea, diammonium phosphate and potassium chloride. The amount of chemical fertilizers for each treatment was unified, which was N 150 kg ha^−1^, P_2_O_5_ 120 kg ha^−1^, K_2_O 90 kg ha^−1^. All of the diammonium phosphate and potassium chloride should be applied as basal fertilizer, while 50% urea was applied as basal fertilizer, and 50% as topdressing at crop filling stage.

**Table 3:** The specific measures for different tillage treatments.

Tillage treatments	Specific measures
Continuous conventional moldboard-tillage (CT)	The moldboard-tillage treatment consisted of mouldboard plowing to a depth of about 20–25 cm, and rotary blade interacted with soil to achieve pulverization with no crop residues after crop harvest
Sub-soiling/moldboard-tillage/sub-soiling tillage(ST)	Sub-soiling tillage and moldboard-tillage were rotated after crop harvest, moldboard-tillage after wheat harvest, sub-soiling after maize harvest. Sub-soiling tillage ploughed the soil to a depth of 30–35 cm with 40–60 cm width between the ploughing strips by sub-soiling shovel, and the maize residues were smashed to cover the surface of the soil
No-tillage/sub-soiling/no-tillage(NT)	No soil tillage is adopted in no-tillage practice, and the wheat residues were smashed to cover the surface soil. No-tillage and sub-soiling were rotated after crop harvest, the no-tillage after wheat harvest, sub-soiling tillage after maize harvest, and crop residues were smashed to cover the surface of the soil after crop harvest to prevent damage to soil structure

### Sampling and measurement methods

Disturbed and undisturbed soils were sampled at depths of 0–10 and 10–20 from each experimental site with a 10-cm diameter soil corer and cutting ring, and the soil aggregate stability, moisture content, organic matter and soil temperature were measured during the maize harvest period (late September 2019). Three soil samples were taken in each experimental plot. We have minimised soil disturbance to avoid disrupting the soil aggregate structure during collection and transportation. Soil microstructure features were characterized by FEI Q45 SEM produced by the American FEI company. When conducted the SEM experiment, air-dried soil samples were coated with an ion sputter instrument to obtain better flat surface, and then microstructure characteristics were observed at the voltage of 10 kV with 1000 magnification times.

Maize yield was determined at 15% grain moisture content by methods of sampling and investigation. Nine experimental plots with an area of about 297 square meters were harvested by hand at maize maturity, and the effective panicle number of each plot was counted. Fifteen representative plants were randomly selected for each treatment, and the number of kernels per spike was counted. The dried maize kernels were at 105 °C dry for 48 h to ensure that the moisture content of maize grain yield was controlled below 15%, 1000-kernel weight of maize was measured by weighing method, and finally the theoretical yield of grain was calculated. Soil pH value was measured by the potential method (soil water ratio 2.5:1). The content of clay and silt was measured by pipette method, and soil bulk density and water content were determined by using cutting ring method and drying method at 105 °C^60,61^. Soil organic carbon (SOC) was measured by the rapid dichromate oxidation method and total nitrogen was measured according to Kjeldahl method^62,63^. Soil temperature of 5, 10, 15, 20, 25 cm soil layer was determined by WNG-11 Right-angle geothermometer, and the average of the soil temperature monitoring values for 3 consecutive days was taken as the soil temperature for this period. The stability and size distribution of soil aggregates at 0–20 cm soil layer were measured by wet-sieve and dry-sieve methods^43,64^. The aggregate content fraction > 0.25 mm (*R*_0.25_), MWD, GMD and fractal dimension (*D*) were calculated as Eqs. 1, 2 and 3, respectively^65–67^.

1 $${\text{MWD}} = \frac{{\sum\limits_{i = 1}^{n} {\left( {\overline{x}_{i} w_{i} } \right)} }}{{\sum\limits_{i = 1}^{n} {w_{i} } }},$$

2 $${\text{GMD}} = \exp \left( {\frac{{\sum\limits_{i = 1}^{n} {w_{i} \ln \overline{x}_{i} } }}{{\sum\limits_{i = 1}^{n} {w_{i} } }}} \right),$$

3 $$\frac{{M\left( {r < \overline{x}_{i} } \right)}}{{M_{T} }} = \left( {\frac{{\overline{x}_{i} }}{{x_{\max } }}} \right)^{3 - D} ,$$

where n denotes the number of aggregate size fractions, $\overline{{x_{i} }}$ is the mean diameter of aggregates retained in the *i*th sieve, *W*_*i*_ is the weight of aggregates retained in the *i*th sieve, *M*(*r* ≤ *x*_*i*_) is the weight of aggregates with a fraction diameter less than or equal to *x*_*i*_, and *CT* is the gross weight of aggregates.

### Statistical analysis

The data were processed and analyzed by Microsoft Excel 2013 and SPSS22.0. Figure generation was performed by using SigmaPlot12.5. One-way analysis of variance of experimental data was conducted through SPSS22.0 and the least significant range (LSD) method was used for multiple comparison, *P* < 0.05 indicating significant level.
